# Orbito-sinal foreign body

**DOI:** 10.4103/0301-4738.71710

**Published:** 2010

**Authors:** Arathi Simha, Mary John, Ruby Rita Albert, Thomas Kuriakose

**Affiliations:** 1 Department of Ophthalmolgy, Christian Medical College, Vellore, India; 2 Department of Otolaryngorhinology and Head and Neck Surgery, Christian Medical College, Vellore, India; 3 Department of Otolaryngorhinology and Head and Neck Surgery, Christian Medical College, Vellore, India; 4 Department of Ophthalmolgy, Christian Medical College, Vellore, India

**Keywords:** Foreign body, maxillary, orbit, paranasal, sinus, stick, wooden

## Abstract

Perforating injuries of the orbit involving the paranasal sinuses are uncommon. We report a case in which a large wooden foreign body lodged in the posterior orbit and maxillary sinus was surgically removed by a combined approach by ophthalmologists and ear, nose and throat surgeons.

Management of foreign bodies lodged in the orbit, paranasal sinuses and anterior cranial fossa varies according to their size, shape, composition, location and wound of entry.[1] We report a case of an intra-orbital wooden foreign body penetrating into the maxillary sinus and its management. MEDLINE search using the words “wooden”, “foreign body”, “orbit”, “paranasal”, “maxillary”, “sinus”, “stick” showed only one other similar report[2] in English literature.

## Case Report

An 18-year-old male presented to us with a 40-day history of injury to the right lower lid below the punctal region while diving in a pond. The wound was sutured immediately by his local doctor. The wound did not heal completely and there was persistent discharge, pain and swelling. Twenty-three days later, he gave history of sneezing out a piece of wood. The patient was referred to us from a neighboring country for further management after Computed tomography (CT) scan revealed a foreign body. He had received a course of oral antibiotics for 10 days following the trauma.

**Figure 1:**
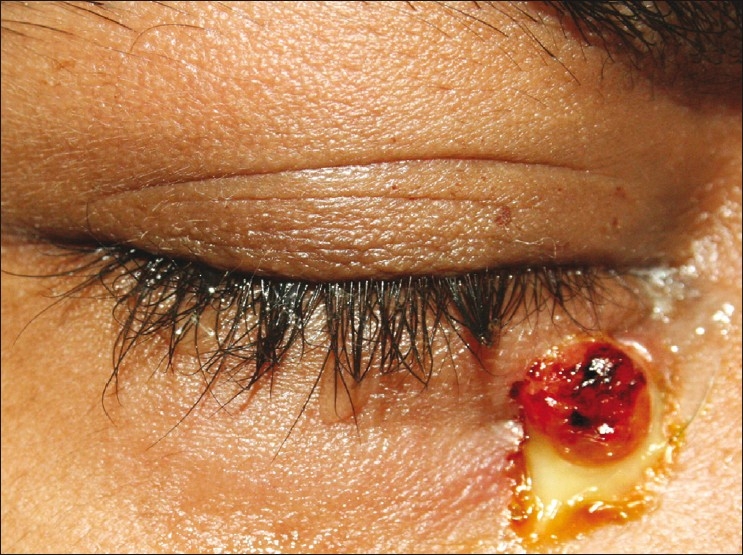
Clinical photograph showing fistula with purulent discharge at the site of wound of entry of the foreign body

On examination, there was a fistula with purulent discharge inferior to the medial end of the right lower lid [Fig. 1] with surrounding erythema, edema and fullness of the cheek. Best corrected visual acuity (BCVA) in the right eye was 20/20. There was a 4-mm proptosis with limitation of elevation and adduction, and congestion of the inferior bulbar and forniceal conjunctiva. Intraocular pressure (IOP) was 18 mm Hg by Goldmann’s applanation tonometry. Rest of the anterior segment and fundus examination was normal. The left eye was normal with BCVA 20/20 and IOP 8 mm Hg.

**Figure 2:**
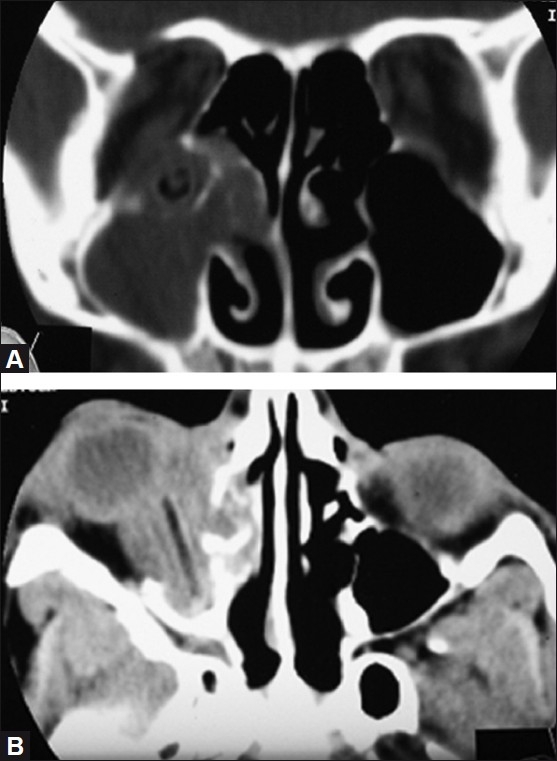
(A) Coronal non-contrast computed tomography (CT) scan showing the foreign body piercing the floor of the orbit into the maxillary sinus. (B) Axial non-contrast CT scan showing the entire extent of the foreign body

CT scan showed a well-defined cylindrical foreign body along the inferomedial right orbital wall, piercing the floor of the orbit posteriorly close to the orbital apex and projecting into the maxillary sinus [Fig. 2A, Fig. 2B]. Thickened soft tissue was seen surrounding the foreign body, suggestive of inflammation/cellulitis. The medial and inferior recti and the inferior oblique could not be distinguished separately. The optic nerve was normal throughout. Maxillary and ethmoid sinuses were opaque.

**Figure 3:**
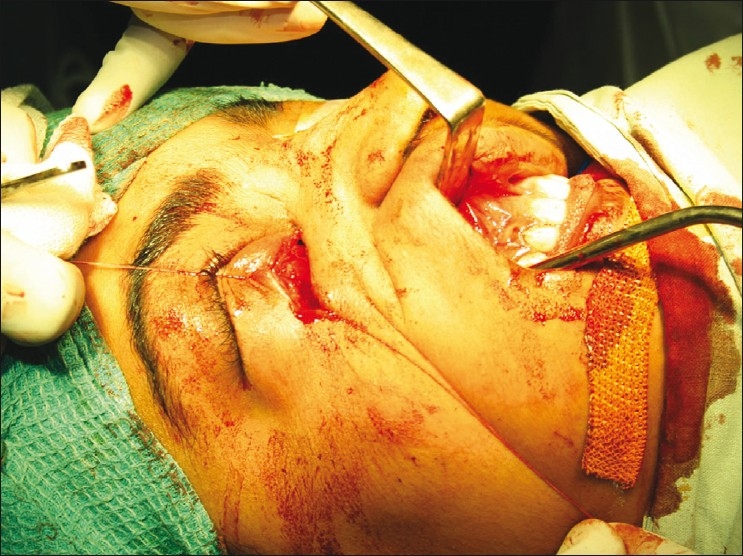
Intra-operative photograph showing combined approach— sublabial and inferomedial orbitotomy incisions

Pus was cultured from the wound but did not grow any organisms. A combined anterior transcutaneous inferomedial orbitotomy and Caldwell-Luc procedure was done under general anesthesia with the ear, nose, throat (ENT) surgeons [Fig. 3]. Oral antibiotics (amoxicillin trihydrate 875 mg + clavulinic acid 125 mg) were started preoperatively.

The maxillary sinus was first opened through a sublabial incision. There was purulent discharge on opening the sinus which was sent for culture. Edematous, inflammed and unhealthy mucosa of the sinus was removed, following which the posterior end of the foreign body was seen penetrating through the posterior part of the roof and protruding into the sinus. Gentle manipulations to remove the foreign body were unsuccessful.

**Figure 4:**
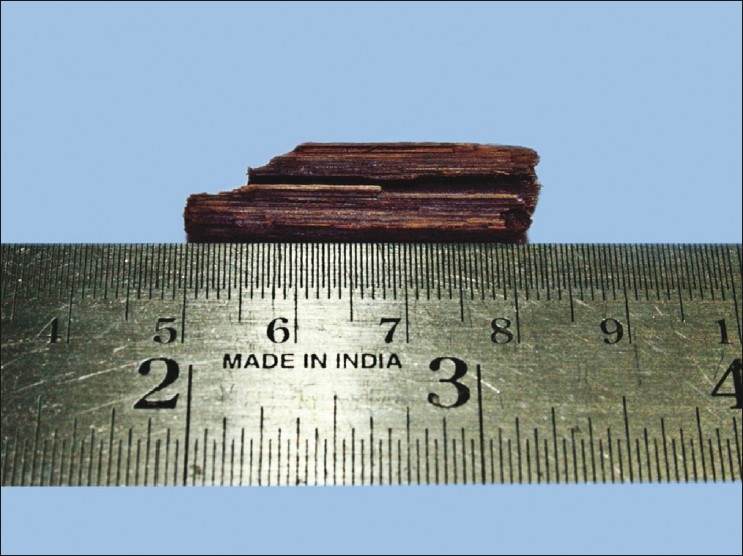
Foreign body, wooden stick removed from the orbit and maxillary sinus

An inferomedial orbitotomy was done through a skin incision along the inferomedial orbital margin [Fig. 3] that included an elliptical area around the fistula. Dissection was carried out posteriorly around the fistula into the region where the foreign body was expected. Intermittent manipulation of the foreign body from the sinus end by the ENT surgeons during dissection significantly aided in localization. On completing the dissection, the foreign body, a wooden stick 3.5-cm long and 0.8 cm in diameter, was pulled out through the orbit [Fig. 4].

Uncinectomy and middle-meatal antrostomy was done endoscopically to aid postoperative drainage of the orbit and maxillary sinus into the nasal cavity. Hemostasis was achieved and the maxillary sinus packed with BIPPS (bismuth iodine paraffin paste) pack. The pack was brought out into the nasal cavity though the antrostomy and was used to pack the nasal cavity also. The sublabial and orbitotomy incisions were sutured.

Postoperatively, the patient received intravenous antibiotics (amoxicillin 1000 mg + clavulinic acid 200 mg) twice daily for two days awaiting culture results, oral non-steroidal anti-inflammatory drugs, and antacids. The culture grew *Escherichia coli* and *Enterobacter* species resistant to amoxicillin and clavulanic acid, but sensitive to ciprofloxacin. The antibiotic was changed to intravenous ciprofloxacin (400 mg twice daily) for three days followed by oral ciprofloxacin (500 mg twice daily) for a week. The postoperative period was uneventful.

**Figure 5:**
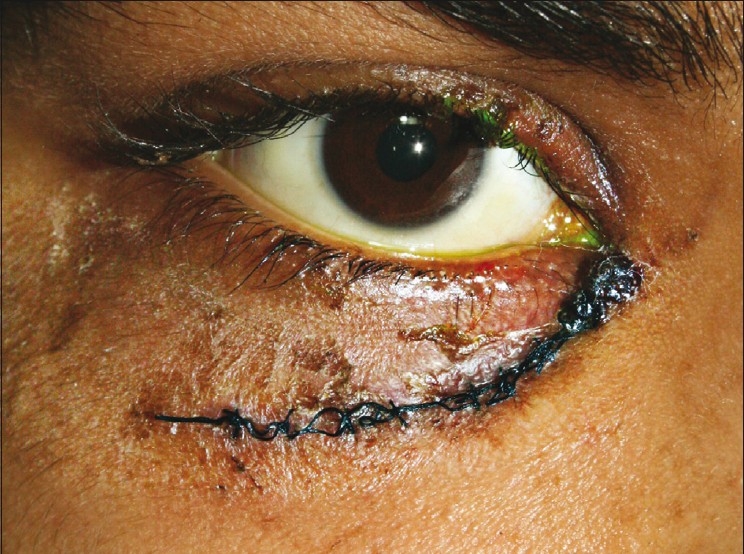
Clinical photograph of the patient one week postoperatively

At review after one week [Fig. 5], BCVA was 20/20, with reduction of proptosis. Patient was orthotropic in primary position with minimal restriction of elevation and mild diplopia in extreme upgaze. The wound was healthy and the sinuses showed no evidence of infection. IOP was 12 mmHg and the rest of the ocular examination was normal except for minimal congestion in the inferior bulbar conjunctiva and fornix [Fig. 5]. Patient wanted to review further only if he had problems, since he was from a neighbouring country and it would be difficult for him to return for a review.

## Discussion

In penetrating injuries of the orbit, imaging has a key role in ruling out intra-orbital foreign bodies, as well as in localization. Wooden foreign bodies can be missed in spite of CT scan since dry wood can be isointense with orbital fat.[3] Magnetic resonance imaging is probably the imaging of choice. However, in our case the foreign body was demonstrated on CT. Foreign bodies that traverse the orbit and the maxillary sinus can be removed transorbitally or though the maxillary sinus, either endoscopically or by opening the sinus. Jagannath *et al*.[2] have reported a large metallic foreign body lodged in the infratemporal fossa, maxillary antrum and floor of orbit removed through the maxillary antrum.

As the nature (wood) of the foreign body and the delayed presentation could cause adherence to surrounding tissues, we preferred combined open approach. Gentle manipulation of the maxillary sinus end of the foreign body was very helpful in determining its exact location, proper dissection and separation from the surrounding adherent tissues. Though the major portion of the foreign body was in the orbit, the maxillary sinus was opened first to avoid contamination of the orbital tissues by the purulent discharge.

In conclusion, factors like location, size, shape and probable composition of the foreign body need to be considered before making a decision on the appropriate surgical plan. Team approach involving ophthalmologists, ENT surgeons and if needed neurosurgeons is necessary. Appropriate antibiotic cover is paramount in infected cases.
